# Malaria parasite species composition of *Plasmodium* infections among asymptomatic and symptomatic school-age children in rural and urban areas of Kinshasa, Democratic Republic of Congo

**DOI:** 10.1186/s12936-021-03919-4

**Published:** 2021-10-02

**Authors:** Sabin S. Nundu, Richard Culleton, Shirley V. Simpson, Hiroaki Arima, Jean-Jacques Muyembe, Toshihiro Mita, Steve Ahuka, Taro Yamamoto

**Affiliations:** 1 Department of International Health and Medical Anthropology, Institute of Tropical Medicine, Nagasaki University, Nagasaki, Japan; 2 Graduate School of Biomedical Sciences, Nagasaki University, Nagasaki, Japan; 3 Program for Nurturing Global Leaders in Tropical and Emerging Communicable Diseases, Nagasaki University, Nagasaki, Japan; 4 Institut National de Recherche Biomédicale, Kinshasa, Democratic Republic of Congo; 5 Division of Molecular Parasitology, Proteo-Science Center, Ehime University, Ehime, Japan; 6 Department of Tropical Medicine and Parasitology, Faculty of Medicine, Juntendo University, Tokyo, Japan

**Keywords:** *Plasmodium*, Malaria, School-age children, Democratic Republic Congo

## Abstract

**Background:**

Malaria remains a major public health concern in the Democratic Republic of Congo (DRC), and school-age children are relatively neglected in malaria prevalence surveys and may constitute a significant reservoir of transmission. This study aimed to understand the burden of malaria infections in school-age children in Kinshasa/DRC.

**Methods:**

A total of 634 (427 asymptomatic and 207 symptomatic) blood samples collected from school-age children aged 6 to 14 years were analysed by microscopy, RDT and Nested-PCR.

**Results:**

The overall prevalence of *Plasmodium* spp. by microscopy, RDT and PCR was 33%, 42% and 62% among asymptomatic children and 59%, 64% and 95% in symptomatic children, respectively. The prevalence of *Plasmodium falciparum, Plasmodium malariae* and *Plasmodium ovale* spp. by PCR was 58%, 20% and 11% among asymptomatic and 93%, 13% and 16% in symptomatic children, respectively. Among *P. ovale* spp., *P. ovale curtisi*, *P. ovale wallikeri* and mixed *P. ovale curtisi* + *P. ovale wallikeri* accounted for 75%, 24% and 1% of infections, respectively. All *Plasmodium* species infections were significantly more prevalent in the rural area compared to the urban area in asymptomatic infections (p < 0.001). Living in a rural as opposed to an urban area was associated with a five-fold greater risk of asymptomatic malaria parasite carriage (p < 0.001). Amongst asymptomatic malaria parasite carriers, 43% and 16% of children harboured mixed *Plasmodium* with *P. falciparum* infections in the rural and the urban areas, respectively, whereas in symptomatic malaria infections, it was 22% and 26%, respectively. Few children carried single infections of *P. malariae* (2.2%) and *P. ovale* spp. (1.9%).

**Conclusion:**

School-age children are at significant risk from both asymptomatic and symptomatic malaria infections. Continuous systematic screening and treatment of school-age children in high-transmission settings is needed.

**Supplementary Information:**

The online version contains supplementary material available at 10.1186/s12936-021-03919-4.

## Background

Despite a widespread reduction in malaria-associated morbidity and mortality in the past decade, malaria remains a major public health problem in sub-Saharan Africa. In 2018, an estimated 228 million cases of malaria resulting in 405,000 deaths occurred worldwide, of which 93% of cases and 94% of deaths occurred in Africa [1].

In sub-Saharan Africa, the vast majority of malaria cases are caused by *Plasmodium falciparum*, with this parasite responsible for 99.7% of all cases recorded in the region in 2018 [1]. *Plasmodium malariae* and *Plasmodium ovale* spp. are thought to be relatively uncommon [2–4] with a prevalence varying between 1 and 17% [2, 5–7]. *Plasmodium malariae* and *Plasmodium ovale* spp. frequently occur as mixed infections with other parasites, which can lead to underestimation of their true prevalence [2, 8–12].

*Plasmodium malariae* infection frequently results in low parasitaemia and commonly occurs in mixed infections with the more common *P. falciparum* or *Plasmodium vivax* [3, 13]. Infections with *P. malariae* may remain asymptomatic for long periods, but it can cause chronic nephrotic syndrome leading to mortality [14–19].

*Plasmodium ovale wallikeri* and *P. ovale curtisi* are responsible of benign tertian malaria, rarely causing severe malaria [20]. These species may, however, cause jaundice, severe anemia, and pulmonary impairments [21], and even death if there is a delay in management [22, 23]. The two species of *P. ovale* spp. display differences in morphology, clinical characteristics, laboratory parameters and genetics [5–7, 9, 12, 24–27].

*Plasmodium vivax* has occasionally been identified among populations in sub-Saharan Africa [28–31], but is considered uncommon due to the high proportion of Duffy negativity amongst the local populations of west and central Africa [32, 33].

The four species commonly found in sub-Saharan Africa, *P. falciparum*, *P. malariae. P. ovale wallikeri* and *P. ovale curtisi* share overlapping ranges and vectors. They are often found infecting the same human populations at the same time, and mixed infections in individual hosts are common. Currently, there is relatively little known about the consequences of their within-host or within-population interactions, and more baseline data regarding their relative prevalence in populations with varying malaria transmission dynamics is required. Furthermore, there is a lack of data regarding the clinical outcomes of mixed species versus single species infections.

For malaria case management and control, rapid diagnostic tests (RDTs) and microscopy are the most widely used diagnostic tools that inform treatment [34]. PCR, whilst more sensitive, is relatively expensive and technically challenging, and so is rarely used as a point-of care diagnostic in endemic areas [35]. Lately, the popularity of RDTs has increased as they are cost-effective and provide an easy-to-use alternative to microscopy [36], which requires skilled microscopists to be optimally effective [37, 38]. However, RDTs are only sensitive and specific for parasitaemia above 200 parasites per µl blood [39] and the most widely used are those that detect *P. falciparum* histidine-rich protein 2 (*Pf*HRP2) [1]. The low sensitivity and specificity of both *Pf*HRP2-based RDTs and microscopy, and a lack of experienced microscopists may result in poor malaria parasite detection [2, 40]. Moreover, the prevalence of non-falciparum parasites may also be underestimated, especially when in co-infections with *P. falciparum* [2, 8–11]. PCR remains the most sensitive method for detecting non-*falciparum* species, even in cases of a very low parasitaemia [40].

Regarding malaria burden, in high transmission sites, symptomatic malaria occurs most often in children under five years old, whereas asymptomatic infections generally occur in older people, school-age children and adults that have acquired immunity against the disease due to repeated exposure [41–44].

School-age children are not usually covered by household-based cluster surveys and/or malaria interventions and so represent an untreated demographic that may harbour a significant parasite reservoir [45–50], thus posing a major challenge for malaria control, surveillance, and elimination strategies [45, 51–53]. Even though school-age children rarely develop complicated forms of malaria, chronic infection among this group is an important major contributor to pathology, including anaemia, and thus may have profound consequences for neuro-cognitive development and educational achievement including increased absenteeism, poor school performance, and cognitive disorders [54–59].

The Democratic Republic of Congo (DRC) is the second most malaria-affected country in the world after Nigeria, and accounts for 12% and 11% of all estimated malaria cases and deaths worldwide, respectively [1]. About 97% of its inhabitants live in perennial malaria transmission zones, in which transmission occurs for 8 to 12 months yearly [60]. In DRC, malaria is still the major cause of morbidity and mortality, accounting for more than 44% of all outpatient visits, and for 22% of deaths among children under 5 years [60]. *Plasmodium falciparum* is the most prevalent malaria parasite species and is responsible for most severe cases [1, 60]. *Plasmodium malariae* and *P. ovale* spp. are uncommon and are mostly observed in co-infections with *P. falciparum* [3]. Of the two *P. ovale* species, only *P. ovale wallikeri* has been identified in the country so far [24]. Reports of the presence of *P. vivax* are rare [30, 61].

Here, the burden of malaria among asymptomatic and symptomatic school-age children living in rural and urban areas of Kinshasa/DRC was assessed and the distribution of *Plasmodium* species in rural areas compared to urban areas was investigated in order to inform the design and employment of school-based control interventions for malaria in this underserved population.

## Methods

### Study design

A cross-sectional study was undertaken between October and November 2019 among school-age children aged 6 to 14 years in Kinshasa, DRC.

### Study area and study population

**Fig. 1:**
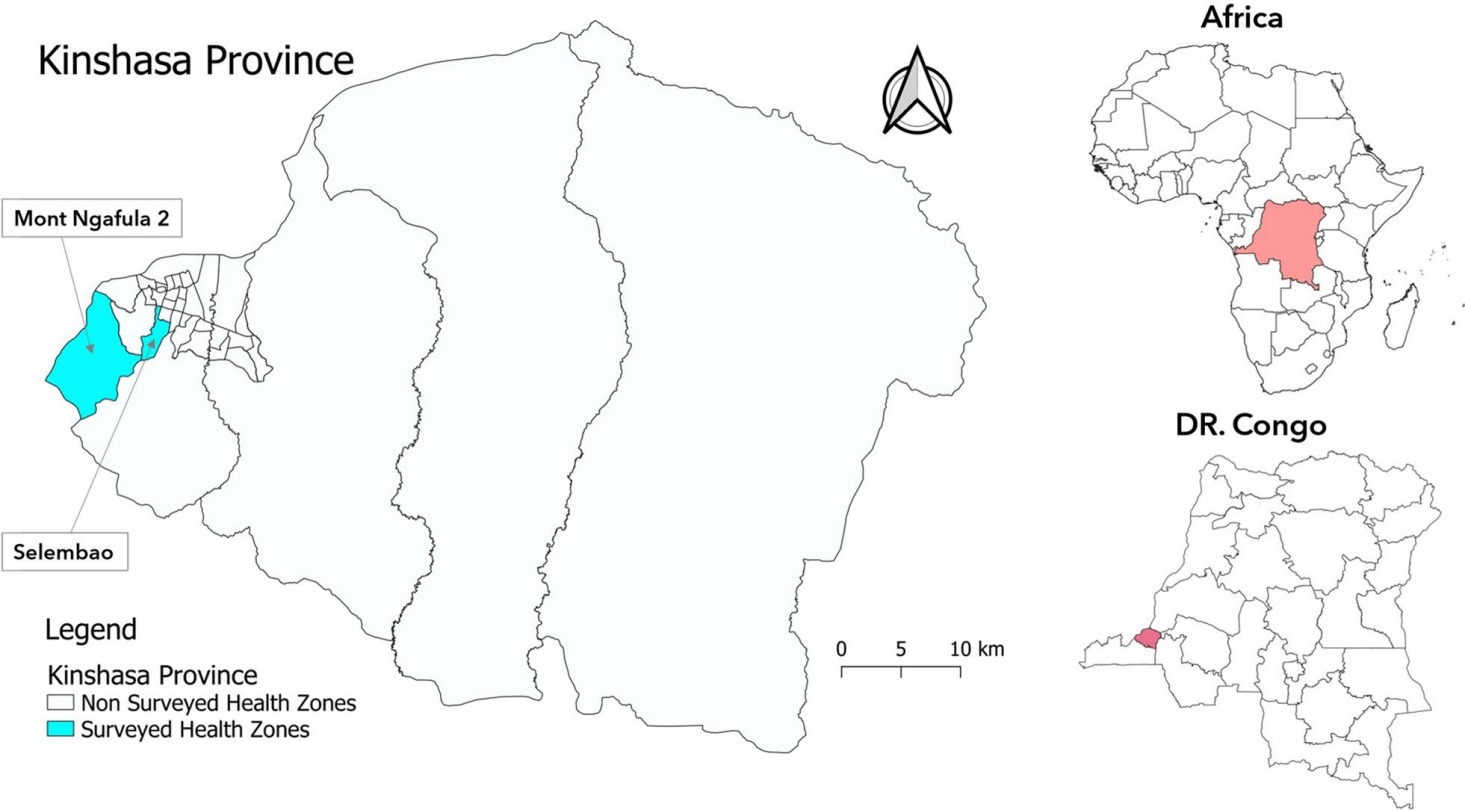
Selection sites

The study was conducted at primary schools and health facilities in the rural area of Mont-Ngafula 2 Health Zone (HZ) and the urban area of Selembao HZ in Kinshasa city (Fig. 1). Selembao HZ is classified as an area at moderate risk while Mont-Ngafula 2 is an area at high risk of malaria [62].

In selected schools, we included all children aged 6 to 14 years with body temperatures less than 37.5 °C during a physical examination and who did not have malaria-related symptoms (including fever, headache, fatigue, chills, nausea, vomiting) in the 2 weeks prior to the survey.

In selected health facilities, we included all outpatient children aged 6 to 14 years who exhibited fever and/or malaria-related symptoms (headache, fatigue, chills, nausea, vomiting) within the three days prior to medical consultation and who had not taken anti-malarial drugs prior to the consultation.

### Sampling and sample size determination

Both hospital-based and school-based surveys were conducted. A two-stage stratified cluster sampling protocol was performed to select two health zones (HZ) among 35 within Kinshasa and their constituent health facilities and primary schools. The sample size was determined using the standard statistical formula n = (Z^2^p (1 − p)/d^2^) considering 95% confidence interval, 50% estimated prevalence and 5% precision. Based on this assumption, 634 school-age children (427 asymptomatic and 207 symptomatic) were included in this study.

### Data collection

In schools, two visits were necessary for sample collection. The first visit consisted of the selection of primary classrooms of each primary grade followed by the distribution of written consent forms to all children belonging to the selected classroom for their parents/guardians. On the second visit, after obtaining written consent from parents/guardians, information related to gender, age, and body temperature were recorded and a physical examination carried out. An interview was conducted for asymptomatic school-age children from 4 to 6th primary grades using a semi-structured questionnaire to record information related to the history of fever and malaria-related symptoms and treatment; socio-demographic status of parents (parent marital status, number of father’s partners, father’s education level, mother’s education level, family size) and information related to the use of mosquito bed nets and/or indoor insecticide spray within households.

The questionnaire was written in English by the research team, and later translated into French (official language). When children were unable to give correct answers to certain questions (questions related to the education level of parent, marital status), the question(s) were addressed to parents through telephone calls.

In health facilities samples were collected day-by-day depending on health facility attendance. Malaria-related symptoms and information related to gender, age, and body temperature were recorded followed by physical examination in health facilities after obtaining consent from outpatient parents/guardians.

### Malaria rapid diagnostic test (RDTs)

An experienced laboratory technician collected approximately 5 μl finger-prick blood from each child to perform RDT. The CareStart™ Malaria *Pf* (HRP2) Ag RDT (RMOM-02571) was used for the qualitative detection of malaria histidine-rich protein 2 in whole blood according to the manufacturer’s instructions. The device remained intact for 20 min at room temperature, and the result was registered accordingly.

### Microscopy of Giemsa's solution-stained blood films

Thick and thin blood smears were made on the same slide in schools) and health facilities. Slides made in schools were sent to the University Hospital, Kinshasa University for reading. Slides were stained for 10 min with 10% Giemsa and washed with distilled water and air-dried. Slides were read by two independent experienced laboratory technicians to determine the *Plasmodium* species and parasitaemia. The asexual forms were counted with reference to 200 leukocytes. In the case that fewer than 100 parasites were counted against 200 leukocytes, the count continued until 500 leukocytes were observed. Parasite density was calculated based on a total of 8000 leukocytes/μl using the following formula: (Number of parasites counted × 8000)/Number of counted leukocytes. The parasitaemia was obtained by averaging the results of the two independents readers.

In the event of discrepant results, a third laboratory technician was involved for confirmation. The laboratory technicians counted a minimum of 100 consecutive fields in the thick blood film before classifying a slide as negative. All slides prepared in schools were read by the experienced microscopists of the Parasitology Unit of the University Hospital, Kinshasa University.

### DNA extraction

In both schools and health facilities, three capillary blood drops from finger-pricks were collected and spotted onto of Whatman 903™ filter paper (Whatman plc, UK) for PCR analysis. Whatman 903™ filter papers containing blood samples were dried and stored in individual plastic bags containing desiccant and stored at − 20 °C before transportation to Nagasaki University for PCR analysis. All DNA templates were extracted from three punched discs (6 mm in diameter) of blood spots using the QIAamp DNA Mini Kit using the QIAamp DNA Mini Kit® (Qiagen, USA) according to the manufacturer’s instructions. DNA was eluted in 50 μL of the provided buffer.

### Nested-PCR for malaria parasite species typing

A nested PCR using primers targeting the *Plasmodium* mitochondrial cytochrome c oxidase III (*cox3*) gene was performed using the protocol described by Isozumi et al. [63] with minor modifications; in particular, the *P. vivax-*specific primers were redesigned due to concerns regarding the non-specific binding of the originally described primers (Additional file 1: Table S1). PCR products were visualized under UV light on 1.5% agarose gels run at 100 V for 30 min and stained with Gel Red® solution for 30 min. The outer PCR product was diluted 1:100 with sterile water and used as template for inner PCRs. *Plasmodium* species genotyping was performed using *Plasmodium* spp. outer PCR products to separately amplify specific products of *P. falciparum, P. malariae, P. ovale* spp. and *P. vivax*. PCR products were visualized under UV light on 2% agarose gels run at 100 V for 30 min and stained with Gel Red® solution for 30 min. The outer PCR product was diluted 1:100 with sterile water and used as template for inner PCRs.

Seventy one out of 79 *P. ovale* spp. positive PCR products were successfully sequenced using the inner PCR forward (Nst_ovaF) primer. There was insufficient material of the remaining eight samples to make a species-level identification. Sequence data files were analysed using MEGA software. Sequence data from NCBI for reference [*P. ovale curtisi* [(GenBank: HQ712052.1) and *P. ovale wallikeri* (GenBank: HQ712053.1)] were used as references. From the sequence data sets, a phylogenetic tree was generated using the NJ method. According to the phylogenetic tree, the samples were assigned species designations based on clustering with the reference sequences.

### Data management and analysis

Data was double-entered and validated in EPI INFO version 3.5.1 and analysed using STATA version 14.2 (College Station. Texas, USA). Descriptive variables were analysed as proportions (qualitative variables) or by median/mean (continuous variables). Chi-square tests (or Fisher’s exact tests when appropriate) and logistic regression analysis were used to assess associations between independent variables and *Plasmodium* spp*.* infection prevalence. Odds ratios (ORs) and 95% confidence intervals (CIs) were derived. Significance was set at *p* < 0.05.

### Ethical considerations

The study was approved by the Ethics committees of the School of Public Health, Kinshasa University, DRC (Approval number: ESP/CE/042/2019) and the Institute of Tropical Medicine, Nagasaki University (Approval number: 190110208-2). Written informed consent was obtained from children’s parents/guardians and assent from children ≥ 7 years old were sought. The written informed consent document was provided either in French (official language) or Lingala (local language) depending on the parent’s educational background. All malaria positive cases with RDTs were treated according to national malaria diagnosis and treatment guidelines.

## Results

### Description of study population

A total of 634 (210 asymptomatic and 105 symptomatic in the rural area; 217 asymptomatic and 102 symptomatic in the urban area) children aged 6 to 14 years old were included in this study.

Their median (interquartile range) age was 9 (7–11) [asymptomatic: 9 (7–11); symptomatic: 8 (7–9)] in the rural area, and 9 (7–10) [asymptomatic: 8 (7–10); symptomatic: 9 (7–12)] in the urban area. Children aged 6 to 9 years accounted for 59.7% (188/315) [asymptomatic: 50.5% (106/210); symptomatic: 78.1% (82/105)] in the rural area, and 63.3% (202/319) [asymptomatic: 68.2% (148/217); symptomatic: 52.9% (54/102)] in the urban setting; 49.5% (156/315) [asymptomatic: 49.5% (104/210); symptomatic: 49.5% (52/105)] in the rural area, and 51.4% (164/319) [asymptomatic: 54.8% (119/217); symptomatic: 41.1% (45/102)] in the urban setting were females (Additional file 1: Table S2).

### Information relating to sociodemographic characteristics of asymptomatic school-age children and malaria preventive measures

Among 227 (131 in the rural area and 96 in the urban area) asymptomatic school-age children who were interviewed, most of children (≥ 80%) lived together with their parents in both rural and urban areas, 60% had at least one mosquito bed net and 31% slept under a mosquito net the night before the interview in the rural area whereas 28% had at least one mosquito bed net and 25% slept under a mosquito net the night before in the urban area (Additional file 1: Table S3).

### History of last fever and/or malaria-like symptoms

**Fig. 2:**
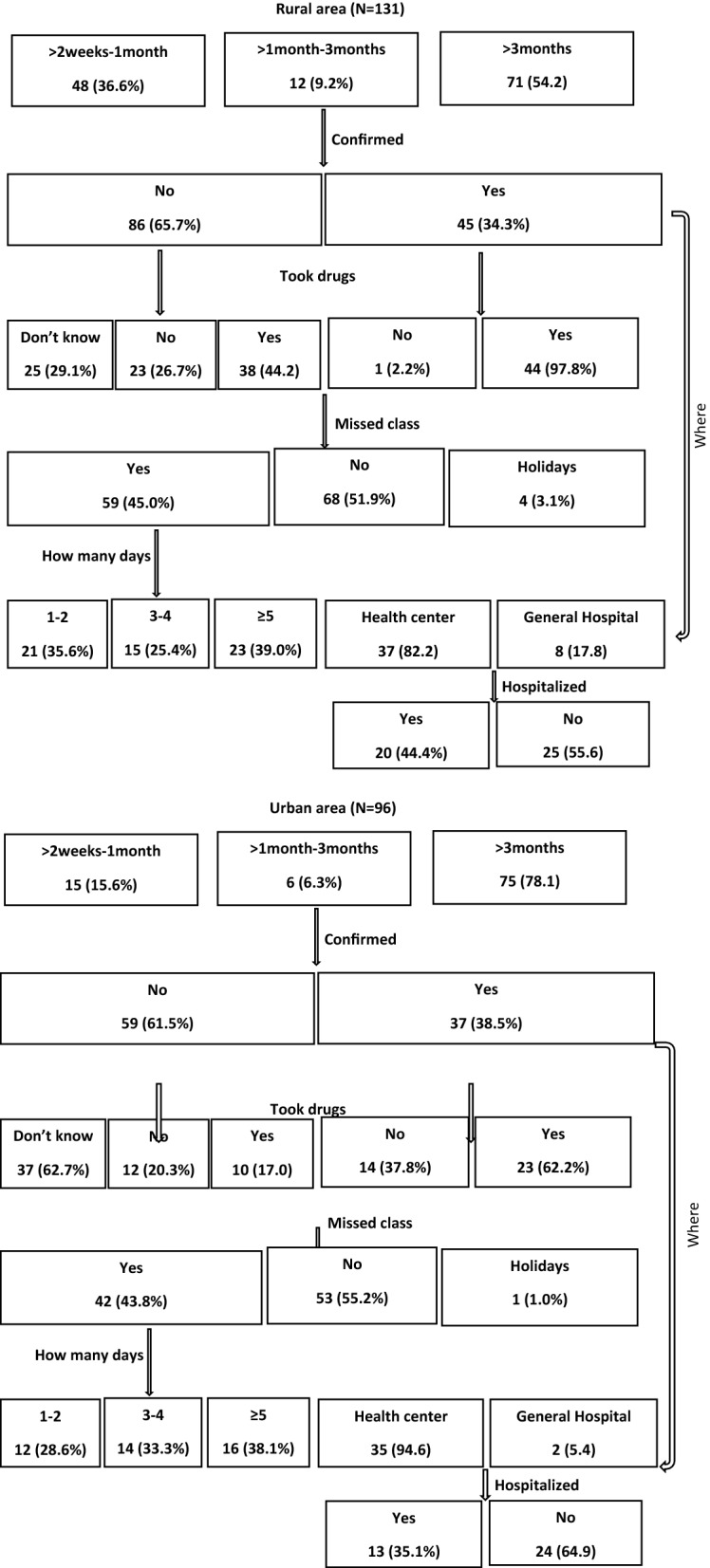
History of malaria-related symptoms among asymptomatic children in the rural (**A**) and urban (**B**) areas

Among 131 children interviewed in the rural area, 54% reported a malaria episode in the three months prior to the survey. Only 34% went to a health facility, and the majority of these (82%) went to a health centre. Forty-four percent of children reported having self-medicated (of which 33% had used sulfadoxine/pyrimethamine (SP) and 11% artemisinin-based combination therapy) given by their parents without a confirmed malaria diagnosis. Fifty-two percent of children missed classes and among these, 39% missed five days or more (Fig. 2).

Among 96 children interviewed in the urban area, a 78% reported a malaria episode in the three months prior to the survey. Only 39% went to a health facility, and the majority of these (95%) went to a health centre. Seventeen percent of children reported having self-medicated with anti-malarial drugs (of which 25% used SP, 25% used artemisinin-based combination therapy and 50% did not remember the drug’s name) given by their parents without a confirmed malaria diagnosis. Forty-four percent of children missed classes and among these, 38% missed five days or more (Fig. 2).

### Symptoms of outpatient school-age children at admission

All 207 children (105 in the rural area and 102 in the urban area) attending health facilities had fever symptoms (100%), followed by vomiting (37%), fatigue (28%) and diarrhoea (23%) in the rural area *versus* headache (72%), fatigue (50%), lack of appetite (49%) and abdominal pain (48%) in the urban setting as major symptoms (Additional file 1: Table S4).

### Prevalence of *Plasmodium* spp. infections by microscopy, RDT and PCR

The overall prevalence of *Plasmodium* spp. was 33%, 42% and 62% among asymptomatic children and 59%, 64% and 94% in symptomatic children by microscopy, RDT and PCR, respectively (Table 1).

**Table 1:** Prevalence of *Plasmodium* spp. infections by microscopy, RDTs and PCR in asymptomatic and symptomatic infections

Technique	Asymptomatic infection (N = 427)	Symptomatic infection (N = 207)
	n (%)	n (%)
Microscopy	140 (32.8)	123 (59.4)
RDT	177 (41.5)	133 (64.3)
PCR	266 (62.3)	196 (94.7)

### Comparison of *Plasmodium* infection prevalence between rural and urban areas among asymptomatic and symptomatic school-age children by PCR

All malaria parasite species were significantly more prevalent in the rural area compared to the urban setting in asymptomatic infections (p < 0.001), whereas in symptomatic infections, *P. malariae* was significantly less prevalent in the rural area compared to the urban setting (7.6 versus 17.7%, p = 0.03). No *P. vivax* infections were observed (Table 2).

**Table 2:** Comparison of *Plasmodium* infection prevalence between rural and urban areas among asymptomatic and symptomatic school-age children

Malaria infections	Asymptomatic infection				p-value	Symptomatic infection				p-value
	Rural area (N = 210)		Urban area (N = 217)			Rural area (N = 105)		Urban area (N = 102)		
	Number	%	Number	%		Number	%	Number	%	
*Plasmodium *species										
*Plasmodium *spp*.*	168	80.0	98	45.2	< 0.001	102	97.1	94	92.2	0.11
*P. falciparum*	161	76.7	87	40.0	< 0.001	101	96.2	92	90.2	0.09
*P. malariae*^a^	66	31.4	19	8.8	< 0.001	8	7.6	18	17.7	0.03
*P. avale* spp.	35	16.7	11	5.1	< 0.001	19	18.1	14	13.7	0.39
Type of *Plasmodium* infection										
Single infection	96	45.7	82	37.8	< 0.001	80	76.2	70	68.3	0.10
Mixed infection	72	34.3	16	7.4	< 0.001	22	21	24	23.5	0.22

^a^Two cases of *P. malariae* by microscopy

Out of 71 children infected with *P*. *ovale* spp., 53 (75%), 17 (24%) and 1 (1%) harboured *P. ovale curtisi*, *P. ovale wallikeri* and mixed *P. ovale curtisi* + *P. ovale wallikeri* infections, respectively. *Plasmodium ovale curtisi* was more frequent than *P. ovale wallikeri* in the rural area (86.0 vs 14.0%) while they were equally distributed in urban area (48% vs 48%) with one child (5%) harbouring a mixed *P. ovale curtisi* + *P. ovale wallikeri* infection (Table 3).

**Table 3:** Distribution of *P. ovale curtis*i and *P. ovale wallikeri* by location, health status, age and gender (N = 71)

	Poc (N = 53)	Pow (N = 17)	Poc + Pow (N = 1)
	n (%)	n (%)	n (%)
Location			
Rural	43 (86.0)	7 (14.0)	0 (0.0)
Urban	10 (47.6)	10 (47.6)	1 (4.8)
Health status			
Asymptomatic	33 (76.7)	10 (23.3)	0 (0.0)
Symptomatic	20 (71.4)	7 (25.0)	1 (3.4)
Age (years)			
6–9	28 (70.0)	11 (27.5)	1 (2.5)
10–14	25 (80.7)	6 (19.3)	0 (0.0)
Gender			
Female	22 (73.3)	7 (23.3)	1 (3.3)
Male	31 (75.6)	10 (24.4)	0 (0.0)

Poc, *P. ovale curtisi*; Pow, *P. ovale wallikeri*

### *Plasmodium* species composition

Of the 462 malaria positive children, 270 (168 asymptomatic and 102 symptomatic) resided in the rural area and 192 (98 asymptomatic and 94 symptomatic) in the urban setting.

In the rural area, 261 (97%) [161 (96%) asymptomatic and 100 (98%) symptomatic] carried *P. falciparum*, 73 (27%) [66 (39%) asymptomatic and 7 (7%) symptomatic] carried *P. malariae*, and 54 (20%) [35 (21%) asymptomatic and 19 (19%) symptomatic) carried *P. ovale *spp*.* parasites (Table 4, Additional file 2: Figure S1K & L).

**Table 4:** Proportion of Plasmodium species in asymptomatic and symptomatic school-age children by location

	Rural area (N = 270)				Urban area (N = 192)			
	Asymptomatic (N = 168)		Symptomatic (N = 102)		Asymptomatic (N = 98)		Symptomatic (N = 94)	
Type of *Plasmodium* infection								
Single infection	96	57.1	80	78.4	82	83.7	70	74.5
Mixed infection	72	42.9	22	21.6	16	16.3	24	25.5
*Plasmodium* species composition								
*P. falciparum*	89	53.0	79	77.4	73	74.5	68	72.3
*P. malariae*	3	1.8	0	0.0	5	5.1	2	2.1
*P. ovale* spp.	4	2.4	1	1.0	4	4.1	0	0.0
*P. falciparum* + *P. malariae*	41	24.4	3	3.9	9	9.2	10	10.6
*P. falciparum* + *P. ovale* spp.	9	5.4	14	13.7	2	2.0	8	8.5
*P. malariae* + *P.ovale* spp.	0	0.0	0	0.0	2	2.0	0	0.0
*P. falciparum* + *P.malariae* + *P. ovale* spp.	22	13.1	4	3.9	3	3.1	6	6.4

In the urban area, 179 (93%) [87 (89%) asymptomatic and 92 (98%) symptomatic] carried *P. falciparum*, 37 (19%) [19 (19%) asymptomatic and 18 (19%) symptomatic] carried *P. malariae*, and 25 (13.0%) [11 (11.2%) asymptomatic and 14 (18%) symptomatic] carried *P. ovale *spp*.* parasites (Table 4, Additional file 2: Figure S1I & J).

There were 89 (53%), 3 (1.8%) and 4 (2.4%) single infections of *P. falciparum*, *P. malariae* and *P. ovale *spp*.*, in asymptomatic participants in the rural area (Table 4, Additional file 2: Figure S1K), while there were 79 (77%), 0 and 1 (1%) in symptomatic participants, respectively (Table 4, Additional file 2: Figure S1L). There were 73 (75%), 5 (5%) and 4 (4%) single infections of *P. falciparum*, *P. malariae* and *P. ovale*, in asymptomatic participants in the urban area (Table 4, Additional file 2: Figure S1K), while there were 68 (72%), 2 (2%) and 0 in symptomatic participants, respectively (Table 4, Additional file 2: Figure S1J).

There were significantly more infections involving *P. malariae* and *P. ovale *spp*.* in asymptomatic children compared to symptomatic children in the rural area (Table 4, Additional file 2: Figure S1G & H), whereas there were similar rates of carriage of these species in asymptomatic and symptomatic infections in the urban setting (Table 4, Additional file 2: Figure S1E & F).

In the rural area, 94 (35%) [72 (43%) asymptomatic and 22 (22%) symptomatic] (Table 4, Additional file 2: Figure S1C & D), individuals carried mixed infections, whereas in the urban area 40 (20%) [16 (16%) asymptomatic and 24 (26%) symptomatic] carried mixed infections (Table 4, Additional file 2: Figure S1A & B). In the rural setting there was a large difference in the proportion of single and mixed species infections between asymptomatic and symptomatic children, with more mixed species infections observed in asymptomatic children compared to symptomatic children (Additional file 2: Figure S1C & D). However, in the urban region there was little difference in the proportion of single and mixed species infections between asymptomatic and symptomatic children (Additional file 2: Figure S1A & B).

Of 12 *P. malariae* and 11 *P. ovale* spp*.* single infections identified by PCR, six were positive by PfHRP2-based RDT; one child harboured *P. malariae* and five children carried *P. ovale* spp*.* infections.

### Association age/gender with *Plasmodium* species infections

**Fig. 3:**
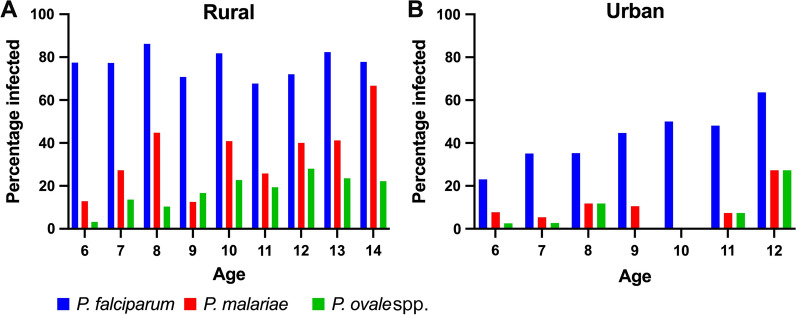
Species composition of *Plasmodium* infections stratified by age in asymptomatic school-age children living in rural (**A** n = 210) and urban areas (**B** n = 214). The number of children aged 13 and 14 years was very low in the urban area and excluded from the figure

Regarding the distribution of the malaria parasite species infecting asymptomatic children by age in the rural and urban areas, the trend of *P. falciparum* did not change with age in the rural setting, while it increased with age in the urban area. *Plasmodium malariae* and *P. ovale* spp. infections were more common in children aged 10 years and above in the rural area (Fig. 3).

In the rural area, asymptomatic children aged 10 to 14 years were significantly more likely to be infected with *P. malariae* (38.5 versus 24.5%, p = 0.03) and *P. ovale* spp. (23.1 versus 10.4%, p = 0.014) than those aged 6 to 9 years. In the urban area, however, older children were more likely to carry *P. falciparum* than younger children (50.7 versus 35.1%, p = 0.029) (Additional file 1: Table S5a). Older children were less likely to harbour single species (33.7 versus 57.6%) and more likely to harbour mixed species infections (44.2 versus 24.5%), (p = 0.02) infections in the rural area, whereas in the urban area older children were similarly likely to harbour single species (46.4 versus 33.8%) and mixed species infections (8.7 versus 6.8%), (p = 0.13) (Additional file 1: Table S6a).

There was no association between gender and any particular *Plasmodium* species infection in either the rural or urban areas (Additional file 1: Table S5a).

In symptomatic children, there was no association between age or gender with particular malaria parasite infections in either the rural or the urban areas except for *P. ovale* spp. which infected younger children more often in the urban setting (4.2 versus 22.2%, p = 0.009) (Additional file 1: Table S5b). Age and gender were not associated with carriage of single compared to mixed infections in either the rural or the urban areas (Additional file 1: Table S6b).

### Predictors of asymptomatic malaria infection

Residence in the rural setting was associated with an increased risk of asymptomatic malaria parasite carriage. There was an approximately five times greater risk of asymptomatic carriage of malaria parasites for children living in the rural, as opposed to urban area (p < 0.001). Other investigated factors were not associated with increased risk of asymptomatic malaria infection (Additional file 1: Table S7).

## Discussion

This study aimed to measure the burden of *Plasmodium* spp. infections including *P. falciparum* and non-*falciparum* single and mixed infections amongst asymptomatic and symptomatic school-age children living in rural and urban areas in Kinshasa, DRC.

Kinshasa, the capital city of DRC, constitutes an urban malaria facies, where malaria prevalence is moderate, with an average of 12% of the population infected at any given time in the city, with increase prevalence variations away from the city centre [60, 64]. Malaria transmission rates are not homogenous throughout the city and depend on the population density and level of urbanization. The prevalence is highest in the more densely populated and less urbanized zones in the periphery [60, 62].

Additionally, malaria infection usually follows a seasonal pattern regulated by mosquito population fluctuations controlled by climate [62, 65]. This study was conducted at the beginning of the rainy season between October and November when conditions of temperature and humidity are favourable for malaria transmission. Temperature, humidity, and rainfall constitute important drivers of mosquito dynamics and malaria risk [65–67].

The overall prevalence of *Plasmodium* spp. was 32%, 41% and 62% among asymptomatic children and 59%, 64% and 95% in symptomatic children by microscopy, RDT and PCR, respectively. The high prevalence found in both asymptomatic and symptomatic children highlights the importance of malaria in this underserved population in Kinshasa.

In asymptomatic children, all *Plasmodium* species infections were significantly more prevalent in the rural area compared to the urban setting. There was a significant difference in malaria prevalence in children living in the rural area as opposed to the urban setting, with the former significantly more likely to be infected with malaria parasites. This finding is in agreement with numerous previous reports, and likely reflects the fact that the ratio of mosquitoes to humans is higher in rural areas than in urban areas [62, 65].

We found that age, generally, was not associated with *Plasmodium* spp. infections. However, there was a significant association between age and asymptomatic *P. malariae* and *P. ovale* spp. infections in the rural area, and *P. falciparum* infections in the urban setting, with children aged 10 to 14 years more infected than those aged 6 to 9 years. Older children were also the group most likely to harbour more single and mixed infections than younger ones. The proportion of children infected with *P. falciparum* remained constant for all ages in the rural area, while it increased with age in the urban setting. *Plasmodium malariae* and *P. ovale* spp. infections increased with age in the rural area while they did not do so in the urban setting. That difference may be due to age-related acquisition of parasite-tolerating immunity [68–70]. It has been shown that in malaria tropical facies, malaria pre-immunity starts building up around 10 years [71]. It may also reflect the relative force of infection of the species, with that of *P. falciparum* being higher than the other two species in the rural setting.

We found a low prevalence of single infections of *P. malariae* and *P. ovale* spp., and a high prevalence of single infection of *P. falciparum* and mixed species infections in both rural and urban areas in agreement with previous reports from Africa [2, 6, 8–12].

Among children infected with *P. ovale* spp., we found 75%, 24% and 1% children harboured *P.ovale curtisi*, *P. ovale wallikeri* and mixed *P.ovale curtisi* + *P. ovale wallikeri*. This finding highlights the fact that *P. ovale curtisi* appears to be more prevalent than *P. ovale wallikeri * [72]. This is the first report of *P. ovale curtisi* in DRC; prior to this study only *P. ovale wallikeri* has been reported [24]. Additionally, this study confirms that *P. ovale curtisi* and *P. ovale wallikeri* can be found in co-infections of the same host [6, 9, 73].

There is a need, perhaps, to focus more attention on the non-falciparum malaria parasites of Africa. The ability of *P. ovale curtisi* and *P. ovale wallikeri* to produce hypnozoites, and the quartan intra-erythrocytic cycle time of *P. malariae* may provide these species with a mechanism for evading the current artemisinin-based combination therapy for uncomplicated malaria [9, 74].

The presence of *P. ovale* spp. and *P. malariae*, co-infected with *P. falciparum*, highlights the impact of those two parasites in asymptomatic and chronic malaria infection. *Plasmodium malariae* and *P. ovale* spp. are not usually associated with severe malaria, but *P. malariae* may be responsible for chronic nephrotic syndrome [14–17, 75, 76], which can be fatal [14–16, 75], and chronic infections that can last for years [15, 76], even after leaving endemic regions [15, 76]. *Plasmodium ovale* spp. is responsible for relapses after months or even years without symptoms due to the presence of hypnozoites [77–83] and it has been shown to cause severe disease and even death on occasion [21–23, 84, 85].

Our data are in agreement with previous reports that show that *P. malariae* is much less likely to be observed in mixed species infections with *P. falciparum* in symptomatic malaria infections when the transmission rate of malaria is high [86, 87]. The reason for this is currently unclear. It is possible that there is a protective effect of mixed infection with *P. malariae* on the severity of the disease caused by *P. falciparum*, perhaps mediated through cross-immunity. It is also possible that in symptomatic *P. falciparum* infections, this species competitively excludes co-infecting species due to increased parasitaemia. This exclusion could result from within-host competition for resources [88], or through host-immune mediated mechanisms in which the innate immune response triggered by the high parasitaemia *P. falciparum* disproportionately affects the less dominant of the species in the co-infection. A further possibility is that the nested PCR methodology used here to determine parasite species may miss the less common of co-infecting species when the disparity between them is large as is likely in symptomatic *P. falciparum* infected patients.

## Conclusion

There is a need to include school-age children in malaria control, surveillance, and elimination strategies. Therefore, a continuous systematic school-based prevention, screening, and treatment of children in high-transmission settings may strengthen malaria intervention measures.

## Supplementary Information

**Additional file 1: Table S1.** Primer sequences and PCR conditions for Plasmodium spp. and Plasmodium genotyping amplification. **Table S2.** Description of study population. **Table S3.** Sociodemographic characteristics of asymptomatic school-age children and malaria preventive measures (N = 227). **Table S4.** Symptoms of outpatient school-age children at admission (N = 207). **Table S5a.** Association between age and gender with asymptomatic malaria infection in rural and urban areas. **Table S5b.** Association between age and gender with symptomatic malaria infection in rural and urban areas. **Table S6a.** Association between age / gender with asymptomatic malaria single and mixed infection in rural and urban areas. **Table S6b.** Association between age / gender with symptomatic malaria single and mixed infections in rural and urban areas. **Table S7.** Predictors of asymptomatic malaria infection (bivariate analysis vs multivariate analysis). Note: COR: Crude odds ratio; CI: Confidence interval; AOR: Adjusted odds ratio

**Additional file 2: Figure S1.** Species composition of *Plasmodium* infections in 98 asymptomatic (Panels A, E and I) and 94 symptomatic (B, F, and J) children in the urban Selembao health zone (HZ), and 168 asymptomatic (Panels C, G and K) and 102 symptomatic (Panels D, H, and L) children in the rural Mont-Ngafula 2 HZ. **The urban Selembao HZ**: Single and mixed infections in asymptomatic (A) and symptomatic (B) children. Malaria parasite species composition in asymptomatic (E & I) and symptomatic (F & J) children. **The rural Mont-Ngafula 2 HZ**: Single and mixed infections in asymptomatic (C) and symptomatic (D) children. Malaria parasite species composition in asymptomatic (G & K) and symptomatic (H & L). Pf = *Plasmodium falciparum*, Pm = *Plasmodium malariae*, Po = *P. ovale wallikeri* and *P. ovale curtisi (“P. ovale”* includes both *P. ovale* species*”).*

## Data Availability

The datasets used and/or analysed during the current study are available from the first author (SSN) upon request.
